# The Palliative Function of Hostile Sexism among High and Low-Status Chilean Students

**DOI:** 10.3389/fpsyg.2017.01733

**Published:** 2017-10-04

**Authors:** Salvador Vargas-Salfate

**Affiliations:** ^1^Facultad de Educación, Universidad Andres Bello, Santiago, Chile; ^2^Department of Psychology, University of Girona, Girona, Spain

**Keywords:** system justification, gender stereotypes, hostile sexism, life satisfaction, children

## Abstract

Previous studies have demonstrated that justifying the social, economic, and political systems is associated with psychological well-being, which has been termed as the palliative function of ideology. However, little research has been conducted on gender stereotypes among children, comparing by socioeconomic status. This study aimed to fill this gap in the system justification literature. We present data from the Chilean version of the International Survey of Children Well-Being (ISCWeB), which was conducted in 2012. We found that the palliative function of gender stereotypes is present among this sample, being qualified by a socioeconomic status by hostile gender stereotype interaction. In other words, the effect on the psychological well-being was observed in low-status, but not in high-status students. These results extend the previous knowledge about the palliative function of the ideology, suggesting why the low-status members of a society actively engage in system justification.

## Introduction

### System Justification Theory and the Palliative Function of the Ideology

The System Justification Theory (SJT; Jost and Banaji, 1994) proposes that people are *actively* motivated to justify the social systems in which they operate. In that sense, people legitimate, bolster, and attribute fairness to the status quo, even if it contradicts their own material interests (Jost and Burguess, 2000; Jost and Banaji, 2004; Jost et al., 2004; Kay et al., 2009; Thorisdottir et al., 2009; Liviatan and Jost, 2014). Previous studies have demonstrated that even in the presence of other alternatives, ceteris paribus, people prefer status quo (Eidelman and Crandall, 2009, 2014; Eidelman et al., 2009, 2010; Jost et al., 2015b).

According to the SJT, there are three not necessarily conscious psychological needs, that explain this theoretical approach (van der Toorn et al., 2010; Hennes et al., 2012). Justifying motives allow individuals to achieve epistemic needs (i.e., certainty and meaning), existential needs (i.e., reducing threat and stress), and relational needs (i.e., sharing values and attitudes). Within this framework, the most studied need is certainty and it has been demonstrated that people prefer control and predictability over randomness (van den Bos, 2009; Tullet et al., 2015).

Justifying social arrangements not only serves to these psychological needs, but also brings some psychological benefits. This argument has been labeled as *the palliative function of the ideology* (Jost and Hunyady, 2002). According to this statement, system justification leads to the buffering of emotional distress in objectively unfair situations. The main involved mechanism is the perception of control, i.e., perceiving social arrangements as unfair or illegitimate reduces subjective well-being, because it depresses perception of control over the environment.

Within SJT, empirical studies have shown that endorsing system-justifying beliefs is associated with psychological well-being (e.g., Harding and Sibley, 2013, Study 1). For example, conservatives, who scored higher on system justification, were highly satisfied with their lives, as compared to liberal people (Napier and Jost, 2008; Butz et al., 2017). However, it has been demonstrated that status and group identity have a moderator role (O’Brien and Major, 2005). In this sense, the association between system justification and psychological well-being (e.g., self-esteem) was observed in low-status individuals who were highly identified with their status group, but not in those who were not identified.

In this article, we explore the *palliative function* of sexism in a representative sample of Chilean students. Three characteristics of our research, taken together, make it valuable to a better understanding of the pervasive presence of sexism, specifically, and system justification, in general. First, SJT, which is the theoretical framework we use as an approach to this phenomenon, has been mainly studied in adult population, with little research among children (e.g., Henry and Saul, 2006; Baron and Banaji, 2009). Second, we focus on sexism, which has concealed less attention in system justification literature as compared with general system justification measures in studying the palliative function of ideology (for notable exceptions, see Napier et al., 2010). And, third, we compare by status variables (i.e., sex and socioeconomic status) because of their theoretical relevance to system justification (Jost et al., 2015a) and the controversial statements derived from this approach (for a discussion, see Caricati and Lorenzi-Cioldi, 2012; Brandt, 2013; Caricati, 2016). In order to fill these gaps, we used an approach based on SJT, including elements from Stereotype Content Model (SCM) and Ambivalent Sexism, because these theories provide a better understanding of the way in which ideologies operate in the specific area of gender relations.

### The Role of Stereotypes and Sexism

The SJT has studied the influence of legitimizing ideologies on system justification (e.g., Jost and Hunyady, 2005; Costa-Lopes et al., 2013), in particular how ideologies such as meritocracy, work protestant ethic, and conservatism, among others, provide an illusory sense that people hold the position in society that they deserve. In this research field, stereotypes have been theorized as a type of ideology that leads to system justification. Particularly, studies have been based on the SCM (Glick and Fiske, 2001a; Fiske et al., 2002; Caprariello et al., 2009; Cuddy et al., 2009), which defines stereotypes in terms of warmth and competence dimensions. The former refers to interpersonal skills, such as friendliness, sincerity, and warmth, and the latter to instrumental performance skills, like capability, confidence, and skillfulness. In addition, SCM proposes that judgments about warmth derive from perceived intergroup competition, so that out-groups that compete for resources with the in-group are targeted as with low warmth. On the other hand, judgments about competence derive from perceived group status, which means that high-status out-groups are targeted as competent. This proposal derives in a two-by-two dimensional space in which an out-group could be perceived in ambivalent terms (i.e., high in one dimension and low in the other).

In SJT research, ambivalent stereotypes have received more attention. Theoretically, the proposed argument is that high- and low-status groups have both positive and negative characteristics. This kind of balance allows individuals to evaluate the social arrangements as fair and legitimate, because both strengths and weaknesses are balanced (Jost et al., 2005; Kay et al., 2007; Kay and Zanna, 2009). The most exemplar research was conducted by Kay and Jost (2003), who across four studies showed that the exposure to the “poor but happy” and “poor but honest” stereotypes led to higher system justification, as compared with non-complementary stereotypes.

Similarly, Glick and Fiske (1996, 1997, 2001b, 2012) and Glick et al. (1997) proposed an approach of prejudice in gender relations. They argued that sexism, which is a gender-based prejudice, could be divided in hostile and benevolent sexism. The former consists in an overtly discrimination against woman. Meanwhile, the latter describes both genders as complementary but different, using a much less aggressive tone, emphasizing the prosocial and intimacy-seeking features of the supposed female personality.

Although sexism is a type of prejudice (Glick and Fiske, 1996) that can be conceptually differentiated from stereotypes (e.g., Devine, 1989; Dovidio and Gaertner, 1999; Stangor, 2009; Amodio, 2014), we include sexism in the discussion about stereotypes, because of two reasons. First, in SJT literature the rationale behind the system-justifying function of sexism refers to the same arguments than complementary stereotypes (e.g., Calogero and Jost, 2011; Connelly and Heesacker, 2012; Napier et al., 2010). Indeed, both gender stereotypes and prejudice are treated as system-justifying ideologies. And, second, some theorists such as Eagly and Mladinic (1989, 1994) have proposed that the cognitive component of attitudes is which defines a stereotype about a group: “when an attitude object is a social group, this cognitive class of response is synonymous with the stereotype about the group” (Eagly and Mladinic, 1989, p. 543). In addition, they proposed that attitudes and stereotypes are highly correlated.

Previous findings have demonstrated that benevolent sexism is transversally and causally associated with gender and general system justification, even among women (Jost and Kay, 2005; Sibley et al., 2007; Laurin et al., 2011). For example, a study conducted by Calogero and Jost (2011, Study 1) showed that women exposed to benevolent sexism scored higher on body shame and self-surveillance than those assigned to the hostile sexism or control conditions. According to the authors, these variables are theoretically related to internalized gender roles. In addition, a series of experimental studies (Becker and Wright, 2011) showed that benevolent sexism is negatively associated with engagement in collective action, but hostile sexism presented a positive relation with this variable. This result suggests that the positive tone presented by benevolent sexism is what explains the passivity observed among women, while hostile sexism is so aggressive that women react to it in form of collective action engagement.

Finally, and in line with the *palliative function of the ideology* hypothesis, benevolent sexism was associated with life satisfaction, mediated by diffuse system justification (Connelly and Heesacker, 2012); and this effect is stronger in more equalitarian societies (Napier et al., 2010). The reason argued for these results is the positive tone emphasized by benevolent sexism, which presents women as possessing positive features, as compared to hostile sexism.

In this article, however, we argue that among children hostile sexism will be associated with life satisfaction. The rationale behind this idea lies on the distinction among stereotypes and personal beliefs proposed by Devine (1989). According to this author, personal beliefs refer to the evaluation of stereotypes, in terms of their appropriateness or adequacy. For that reason, personal beliefs are cognitively and necessarily developed after the acquisition of stereotypes. In addition, children tend to adopt stereotypes at early stages of their development. Then, we hypothesize that children will not have fully developed personal beliefs that reject overt expressions of sexism yet, so that we will be able to identify the *palliative function* of hostile sexism. In addition, a review conducted by Baron and Banaji (2009) showed that children at early stages of development have attitudes coherent with system justification, such as the lack of in-group preferences among low-status groups. In that sense, hostile sexism would operate as a legitimizing ideology, in the same vein than meritocracy, protestant work-ethic, among others (e.g., Jost and Hunyady, 2005; Costa-Lopes et al., 2013), despite its directly aggressive tone. Therefore, the first hypothesis of the article is as follows:

H1: Hostile sexism will be positively related to life satisfaction.

### Status Comparison and SJT

The SJT proposes that system justification is a type of motivation different from self and group justification. Among members of high-status groups, these motivations are complementary. However, among members of low-status groups, system justification contradicts the other motivations (Jost et al., 2015a). In other words, for disadvantaged individuals perceiving the social arrangements as fair and legitimate lead to blame themselves by their position within society, which in turn affects individual and collective self-esteem. Based on this rationale, higher system justifying beliefs are expected among high-status groups. Nevertheless, the strong form of SJT (Jost et al., 2003) has proposed that under certain circumstances low-status individuals are more prone to perceive the social arrangements as fair than high-status individuals. This argument stems from the cognitive dissonance theory (Festinger and Carlsmith, 1959; Festinger, 1962; Chapanis and Chapanis, 1964; Greenwald and Ronis, 1978; Elliot and Devine, 1994; Metin and Metin Camgoz, 2011), which argues that when people are confronted with two contradictory cognitions a dissonance is experienced and, therefore, individuals overcome it by rationalizing one of them. The application of this theory to SJT proposes that low-status people must deal with a cognitive dissonance between ego/group justification and system justification motivations. Under certain circumstances (e.g., in a democratic and highly unequal context, and within a meritocratic culture), the dissonance is highly salient and it is resolved by rationalizing the status quo, so we could expect that low-status individuals should be more motivated to justify the system than high-status individuals, endorsing system justifying beliefs in a higher degree (Jost et al., 2001, 2003; Gaucher and Jost, 2011; Sengupta et al., 2015). However, this proposal has been highly controversial and cross-cultural researches, using representative samples, have found little support for this view (e.g., Caricati and Lorenzi-Cioldi, 2012; Brandt, 2013; Caricati, 2016). In the specific case of gender relations and sexism, an extension of the strong form of SJT imply that endorsing hostile sexism among women is a way to preserve a positive vision about the system-gender relations, via a subjective internalization of a disadvantaged position within society. Indirect evidence for this acceptance of subordination has been observed in the *depressed entitlement* paradigm, that has shown that women have a lower sense of deservingness when asked to rate their own work, as compared with men (Jost, 1997; Blanton et al., 2001; O’Brien et al., 2012).

Nevertheless, empirical studies on sexism and gender stereotypes in Chile propose a slightly different scenario, which is important given that stereotypes are context dependent, so their content vary as the gender relationships change in society (Diekman et al., 2005). Most of these studies have been focused on the validation of the Ambivalent Sexism Inventory (e.g., Mladinic et al., 1998), but some of them have provided comparisons by sex and socio-economic status. For example, Cardenas et al. (2010) found no differences by socioeconomic status in hostile sexism among undergraduate students. A study conducted with a national representative sample (PNUD, 2010) found five social representations of gender relations using cluster analysis, of which two of them are coherent with hostile sexism and comprises 36% of the sample. In these groups, most of the cases classified belong to low-social economic groups, and in the most sexist group, there is a prevalence of men. This is striking, because since the beginning of Michelle Bachelet’s first government in 2006, in Chile there is a strong gender equality agenda, which has promoted pension reforms to achieve a less unequal income distribution by gender, laws against domestic violence, among others (Thomas, 2016). However, there are no longitudinal studies to date in order to compare stereotypes before and after the introduction of this public agenda.

Given these arguments, based on SJT and the specific Chilean context, the second hypothesis is as follows:

H2a: Boys will engage in hostile sexism in a higher degree than girls will.
H2b: High-status students will engage in hostile sexism in a higher degree than low-status students will (SJT hypothesis), or low-status students will engage in hostile sexism in a higher degree than high-status students will (context hypothesis).

Given that ego and group justification are contradictory to system justification among low-status groups, but not among high-status groups (Jost et al., 2015a), it is more likely to found the *palliative effect* of system justification among high-status people (Jost and Hunyady, 2002). The empirical evidence for this argument is mixed. Several studies have indeed found that system justifying beliefs lead to better general psychological well-being among high-status groups (e.g., McCoy et al., 2013), whereas other researches failed to find significant differences by status (e.g., Sengupta et al., 2017), and other group of studies revealed a mixed pattern (e.g., O’Brien and Major, 2005; Rankin et al., 2009; Bahamondes-Correa, 2016; Godfrey et al., 2017). Despite the lack of a meta-analysis that could lead to a better understanding of this phenomenon, O’Brien and Major (2005) proposed that social identification is a moderator among low-status groups, so that among those highly identified the effect of system justifying motives should be negative.

A recent study tested specifically the statements derived from the palliative function of ideology among a student sample (Godfrey et al., 2017). This research found that endorsing system-justifying beliefs longitudinally predicted depressed levels of self-esteem, providing partial support for the statements proposed by SJT (Jost and Hunyady, 2002). However, there are two main differences with our study. First, they used a sample of adolescents from deprived neighborhoods, so we cannot rule out the same pattern of findings among high-status individuals. In our study, on the contrary, we used a sample from heterogeneous social and economic backgrounds. And, second, they use a version of the general system justification scale (Kay and Jost, 2003), instead of a sexism measure, as we do in this article.

Based on the proposed arguments, the third hypothesis is as follows:

H3a: Hostile sexism by sex interaction will predict life satisfaction, so that the effect of hostile sexism will be positive among boys and negative among girls.
H3b: Hostile sexism by socioeconomic status interaction will predict life satisfaction, so that for high-status students the relationship between hostile sexism and life satisfaction will be positive and negative for low-status students.

In sum, the present research aimed to fill the gap in the palliative function of gender stereotypes among children from different status, which is particularly relevant because stereotypes are acquired in the early childhood. Previous researches have studied the differences between children status in system justification (Henry and Saul, 2006), but not the effect on psychological well-being among this population.

We used a national-representative sample from the Chilean version of the International Survey of Children Well-Being (ISCWeB), conducted in 2012. Due to the availability of variables and the scarcity of research in this population, we focused on hostile sexism. We expect to find the same results formerly reported, as stereotypes are formed during the early childhood, even before the possibility to think and reflect about them (Devine, 1989).

## Materials and Methods

### Participants

The data was collected during the second wave of the International Survey of Children Well-Being (ISCWeB), conducted in 2012. The ISCWeb is an international survey conducted in 20 countries, whose aim is to collect data for improving children’s well-being (Children’s World, n.d.). The survey has three versions for 8-, 10-, and 12-year-old children, which correspond with third, fifth, and seventh grades, respectively. These versions contain a similar core questionnaire, but differ in the metrics used for the youngest children (8-year-old), and in inclusion of additional variables for the 10- and 12-year-old versions. The differences between these latter versions are related to the presence of a few items mainly related to money, which were asked to 12-year-old children, but not to 10-year-old children. The rest of the core questionnaires were the same for these two age groups, both in the item phrasing and the metrics. In addition, each local research team developed further measures according to their interests. In Chile, different variables about gender roles and relationships were included in the 10- and 12-year-old versions, so we decided to use them for this article. All the variables used for this research were the same for both versions.

The sample was representative of the three main regions of the country (Metropolitana, Valparaíso, and Biobío), which contain above the 70% of the Chilean population. The design was a stratified and multi-staged sample (Oyanedel et al., 2015). In each region, schools were randomly selected according to stratums defined by their status. Next, in each school third, fifth, and seventh grade classes were randomly selected to participate in the study. Finally, all the students present in the classroom took part in the study. The questionnaire was briefly introduced by a monitor and students completed the instrument by themselves. The survey accomplished all the ethical requirements for a study conducted among children, asking for active consent from school directors, parents, and children.

One thousand six hundred and sixty-five students from 72 schools participated in the selected versions (53.0% in the 12-year-old version; 55.4% boys). The mean age was 11.54-years-old (*SD* = 1.356). According to the socioeconomic status variable, 31.8% belonged to the high status, 40.2% to the medium status, and 28.0% to the low status group.

### Measures

#### Hostile Sexism

This instrument was developed specifically in the framework of the ISCWeB Chilean survey, and consisted in assessing the agreement with the following six items: “To change diapers, to bath, and to feed children are duties of the mother,” “The woman’s most important task is to take care of the house and to cook for her family,” “The man is who must make the most important decisions in the home,” “To clear the table is a woman’s responsibility,” “Women must stay at home and men must go to work,” and “To clean the house is a woman’s duty.” The scale ranged from 1 (“totally disagree”) to 4 (“totally agree”), with higher scores indicating higher endorsing of hostile sexism. A confirmatory factor analysis, which used robust unweighted least squares estimator (ULSMV) because of the ordinal scale, revealed an adequate goodness of fit [^χ^2^^ (9) = 132.889, *p* < 0.001; CFI = 0.981; TLI = 0.968; RMSEA = 0.092] and all the factor loadings were higher than 0.50, providing evidence of the validity of our measure. In addition, reliability, computed using an index that does not assume tau-equivalence (Wang and Wang, 2012), was high (ρ = 0.881).

#### Brief Multidimensional Student’s Life Satisfaction Scale (BMSLSS)

This instrument was developed to assess subjective life satisfaction in students (Seligson et al., 2003) depending on their developmental stage. This scale comprises six items related to satisfaction with several domains (e.g., family life, neighborhood), in a similar vein that other measures used among adult population (e.g., Lau et al., 2005). The scale ranged from 0 (“totally unsatisfied”) to 10 (“totally satisfied”), with higher scores indicating higher life satisfaction. We validated this instrument through a confirmatory factor analysis with the maximum likelihood estimator, deleting the item related to friends, due to its low factor loading. We obtained an adequate goodness of fit [^χ^2^^ (5) = 41.939, *p* < 0.001; CFI = 0.972; TLI = 0.943; RMSEA = 0.067] with all factor loadings higher than 0.45, and an acceptable reliability (ρ = 0.707) for the five-item measure.

#### Socioeconomic Status

We used the School Vulnerability Index (SVI; JUNAEB, 2013), which is a proxy variable to assess socioeconomic status. In Chile, this is a commonly used index that is computed at the school-level, according to the socioeconomic characteristics students. The index is calculated based on the socioeconomic characterization obtained through surveys, and using secondary data, identifying students who belong to families benefited by social policy programs and poor contexts in general (JUNAEB, n.d.). This measure is used by the Chilean government to provide additional resources to the most deprived schools by focusing the social spending. Theoretically, this index ranges from 0 (non-vulnerable students) to 100 (all the students are vulnerable), and lower scores indicate higher socioeconomic status.

## Results

Descriptive statistics are shown in **Table 1**. The hostile gender stereotype mean was 2.26 (*SD* = 0.858), i.e., significantly lower than the middle point of the scale 2.5, *t*(1544) = -10.822, *p* < 0.001. In addition, in the whole sample, the mean of life satisfaction was 8.60 (*SD* = 1.537). Hostile sexism significantly related to the SVI and life satisfaction, in the expected direction. In other words, more sexism endorsed by the students, the more vulnerability (or the less socioeconomic status), and the higher life satisfaction. In addition, the SVI was not significantly associated with life satisfaction. These results provide support for Hypothesis 1 (i.e., hostile sexism will be positive related to life satisfaction), but lead to reject Hypothesis 2b (i.e., hostile sexism will be differentially endorsed by high- and low-status students).

**Table 1:** Matrix correlation.

	*M*	*SD*	1	2	3
(1) Hostile sexism	2.26	0.858	1		
(2) School Vulnerability Index	60.17	22.998	0.255^∗∗∗^	1	
(3) BMSLSS	8.60	1.537	0.124^∗∗∗^	-0.001	1

^∗∗∗^*p < 0.001*

A linear regression with hostile gender stereotype as outcome variable, entering simultaneously the independent variables, was performed, which is shown in **Table 2**. The model was significant, *F*(3,1539) = 73.78, *p* < 0.001, R^2^ = 0.126. The results indicate that boys endorsed more hostile gender stereotype than girls, and the more vulnerability (or the less socioeconomic status), the higher the score in the dependent variable when controlling for age. In a second model, we introduced the SVI by sex interaction simultaneously to the other independent variables, obtaining a significant regression, *F*(4,1538) = 55.50, *p* < 0.001, R^2^ = 0.126. However, the interaction was not significant. These results provide support for Hypotheses 2a (i.e., boys will engage in hostile sexism in a higher degree than girls will), but not for the Hypothesis 2b (i.e., hostile sexism will be differentially endorsed by high- and low-status students).

**Table 2:** Linear regression: hostile sexism.

	Model 1				Model 2			
	*b*	*SE*	*t*	*p*	*b*	*SE*	*t*	*p*
Sex	0.25	0.041	6.02	0.000	0.34	0.114	2.96	0.003
School Vulnerability Index	0.01	0.001	9.66	0.000	0.01	0.001	7.09	0.000
Age	-1.33	0.015	-8.73	0.000	-0.13	0.015	-8.74	0.000
School Vulnerability Index by Sex					0.00	0.002	-0.08	0.404
Constant	3.16	0.192	16.45	0.000	3.11	0.200	15.54	0.000

**FIGURE 1:**
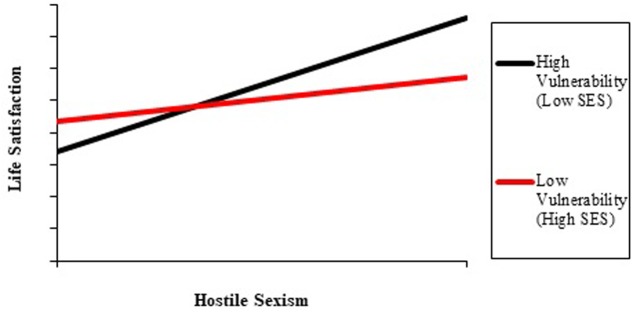
School vulnerability by Hostile sexism interaction.

A linear regression with BMSLSS as the outcome variable, entering simultaneously the independent variables, was performed, obtaining a significant model, *F*(4,1423) = 12.07, *p* < 0.001, R^2^ = 0.033. The results in the **Table 3** showed that there is a positive and significant relationship between hostile sexism and life satisfaction, which is in line with Hypothesis 1, controlling for sex, school vulnerability and age. In a second model, we introduced two two-way interactions (hostile gender stereotypes by sex/school vulnerability) simultaneously to the other independent variables, obtaining a significant regression, *F*(6,1421) = 10.02, *p* < 0.001, R^2^ = 0.046. The sex by hostile gender stereotype interaction was not significant, so we can discard Hypothesis 3a (i.e., hostile sexism by sex interaction will predict life satisfaction), so that the effect of hostile sexism will be positive among boys and negative among girls; but the SVI by hostile gender stereotype was significant. The **Figure 1** shows the simple slope-analysis, which revealed that at low scores in the vulnerability index (or at high socioeconomic status), the relationship between gender stereotype and life satisfaction was not significant (-1SD, *b* = 0.03, *SE* = 0.075, *t* = 0.035, *ns*); but at high levels (or at low socioeconomic status), the reverse was true (+1SD, *b* = 0.32, *SE* = 0.066, *t* = 4.91, *p* < 0.001). These results reject the statement proposed by Hypothesis H3b (i.e., hostile sexism by socioeconomic status interaction will predict life satisfaction, so that for high-status students the relationship between hostile sexism and life satisfaction will be positive and negative for low-status students).

**Table 3:** Linear regression: BMSLSS.

	Model 1				Model 2			
	*b*	*SE*	*t*	*p*	*b*	*SE*	*t*	*p*
Hostile sexism	0.19	0.051	3.79	0.000	-0.14	0.155	-0.89	0.372
Sex	0.06	0.083	0.73	0.466	0.38	0.229	1.66	0.097
School vulnerability	0.00	0.002	-1.89	0.059	-0.02	0.005	-3.54	0.000
Age	-0.15	0.031	-4.79	0.000	-0.15	0.031	-4.78	0.000
Sex by hostile sexism					-0.14	0.096	-1.45	0.147
School vulnerability by hostile sexism					0.01	0.002	3.04	0.002
Constant	10.08	0.416	24.20	0.000	10.68	0.512	20.86	0.000

Given the hierarchical structure of the data, we replicated the previous models using multilevel-linear regressions (Gelman and Hill, 2007) with the restricted maximum likelihood estimator in the **Table 4**. In the first model, we included only the intercept in order to compute the intra-class correlation. In the second model, we added the individual-level predictors (i.e., hostile sexism, age, and sex) and the school-level predictor (i.e., socioeconomic status). Finally, in the third model, we included the interaction terms. All the continuous individual-level predictors were group mean centered, and the school-level predictor was grand mean centered.

**Table 4:** Multilevel-linear regressions: BMSLSS.

	Model 1				Model 2				Model 3			
	*b*	*SE*	*z*	*p*	*b*	*SE*	*z*	*p*	*b*	*SE*	*z*	*p*
Hostile sexism					0.19	0.052	3.59	0.000	0.25	0.080	3.09	0.002
Sex					0.04	0.083	0.52	0.602	0.05	0.083	0.60	0.549
Age					-0.12	0.052	-2.34	0.019	-0.11	0.052	-2.21	0.027
School Vulnerability Index					0.00	0.003	-0.31	0.758	0.00	0.003	-0.36	0.716
Sex by Hostile sexism									-0.12	0.105	-1.18	0.239
School vulnerability Index by Hostile sexism									0.01	0.002	3.13	0.002
Constant	8.57	0.059	145.06	0.000	8.55	0.076	112.42	0.000	8.56	0.076	112.26	0.000
-2Log	-2799.76				-2628.97				-2623.19			
AIC	5605.52				5271.94				5264.38			
BIC	5621.50				5308.78				5311.75			

Model 1 included only the intercept, obtaining an intra-class correlation of 0.054, so that the 5.4% of the dependent variable’s variance is explained by the nested structure of data. Model 2 included the predictors, obtaining a better fit than in Model 1 (Δ-2Log = -170.789, ΔAIC = -333.579, ΔBIC = -312.716). Hostile sexism was a positive predictor of BMSLSS, and age was negatively associated with this variable. Neither sex nor SVI were significant. Model 3, finally, presented a better goodness of fit than Model 2, according to -2Log (Δ = -5.779) and AIC (Δ = -7.559). In accordance to linear regression models, the interaction term between SVI and hostile sexism was significant. The simple slope analysis revealed that the effect of hostile sexism on BMSLSS was stronger in high vulnerable schools (+1SD, *b* = 0.82, *SE* = 0.208, *z* = 3.94, *p* < 0.001) than in low vulnerable schools (-1SD, *b* = 0.45, SE = 0.099, *z* = 4.59, *p* < 0.001).

## Discussion

The purpose of the present research was to extend the study of the *palliative function of ideology* to the children population and to the gender stereotype field. According to SJT, endorsing system-justifying beliefs is associated with general well-being, because the fulfilling of existential, epistemic, and relational needs (Jost and Hunyady, 2002). Nevertheless, little research has been conducted among children, and specifically on this hypothesis (for an exception, see Godfrey et al., 2017). We used a representative sample of Chilean students, who were surveyed in 2012, to demonstrate this palliative effect.

The results partially supported the proposed hypotheses. First, we found that boys engaged in hostile sexism in a higher degree than girls did, in a similar vein than a study conducted among Flemish adolescents (Vandenbossche et al., 2017). Additionally, low-status students engaged more in this stereotype. According to the SJT (Jost and Banaji, 1994), among high-status individuals the ego and group justification motives, on the one hand, and system justification motive, on the other hand, are coherent and complementary, which does not occur in low-status individuals (Jost et al., 2015a). Under certain circumstances, low-status individuals could be more motivated to endorse ideologies that justify the system (Jost et al., 2003) given the necessity to solve the cognitive dissonance among the mentioned justification motives. In our study, we found a mixed pattern, although the more direct status relevant variable for the dependent variable (i.e., sex) contradicted this statement proposed by SJT. These results fit more appropriately with a vision based on the specific context of Chilean culture. According to previous studies, men and low-socioeconomic status individuals endorse a traditionalist gender relationship representation (PNUD, 2010), which is coherent with hostile attitudes toward women.

Second, hostile sexism predicted life satisfaction. This result allows us to observe that the *palliative function of the ideology* hypothesis (Jost and Hunyady, 2002) is confirmed among children using a sexism measure. Most of the literature on this topic has found that benevolent sexism, and not hostile sexism, is associated with psychological benefits (e.g., Connelly and Heesacker, 2012) given that this form of prejudice attributes positive and negative characteristics to women, which leads to an equivalent illusory scenario in gender relations. However, we expected to find a relationship between hostile sexism and life satisfaction, because of the population studied. Stereotypes are acquired during early childhood (Devine, 1989) and thus the conscious control of these should be clearer and evident during adulthood. We hypothesized that children are at an early stage of personal belief development, so that they have less cognitive tools (e.g., ideologies, political positions) to reject overt types of prejudice in a clear way.

Third, the relationship between hostile sexism and life satisfaction was not qualified by sex interaction. Although the endorsement of sexism was higher among boys, as compared to girls, the payoff of this variable did not differ by sex. This is an interesting result, because it allows understanding one of the mechanisms by which social systems are maintained in the long-term. In addition, the interaction between sexism and socioeconomic status predicted life satisfaction, but in a different form as expected, with the effect being stronger among low-status groups. If we consider together the results of both interactions, we found little support for the statement derived from the SJT, which argues that the *palliative effect* of ideology is stronger among high-status individuals, because of the coherence between the ego, group, and system justification motives (Jost and Hunyady, 2002). These results suggest that the hedonic benefits of endorsing beliefs, stereotypes or ideologies that justify the system are more homogeneously distributed than in the proposed theory, which is at odds with recent studies in the field (e.g., Sengupta et al., 2017).

In sum, the present research demonstrated that the *palliative function of the ideology* hypothesis is applicable to children population using a gender stereotype measure, and its differential impact by status.

### Implications

The main contribution of our study is associated with testing the theoretical statements derived from SJT to a sample composed by children, in a similar vein that previous studies (e.g., Henry and Saul, 2006; Baron and Banaji, 2009; Godfrey et al., 2017). According to Devine (1989), in childhood is more likely to observe stereotypes in an explicit form, given that personal beliefs, which are evaluations of stereotypes, are acquired later on development. For this reason, we expected to find that an overt measure of sexism would be associated with well-being. If we consider these results, with the antecedents of the Chilean context, in which social representations about gender relations correspond with traditionalist views, we would propose two alternative hypotheses. First, among high-status individuals, the acquisition of personal beliefs could be earlier in development than low-status individuals, and for that reason we observed a higher endorsing of sexism in this group as compared with high-status individuals. Second, the results reflect the group influence on children, given that studies in Chile, using representative samples, showed a similar pattern in adult population. Although this type of social influence has not been addressed within SJT research field, it is not contradictory with its main statements. This hypothesis would add to this approach a specific causal mechanism involved in the acquisition of stereotypes, prejudice, personal beliefs, and ideologies. In addition to the present findings, we think this is a plausible hypothesis, given that it converges with results from related areas, mainly focused on the transmission of ideology and moral values related to politics (e.g., Sears and Levy, 2003; Tagar et al., 2014). Our study cannot rule out either of these two hypotheses, given that we used cross-sectional data. However, we suspect that both of them could account for part of the phenomenon on study.

Regarding SJT and the palliative function of ideology (Jost and Hunyady, 2002), our results confirm the general pattern predicted by theory, so that endorsing a type of system-justifying belief is associated with well-being. Nevertheless, the comparisons by status led to a rejection of the specific statements proposed by this theoretical approach. Neither sex nor socio-economic status moderated the palliative function in the expected direction. It appears a homogeneous effect by sex, which is the more direct status variable regarding gender relations, and a positive effect among low-status individuals, instead of negative as the theory argues. We hypothesize that considering the Chilean context, in which low-status individuals are involved in traditionalist views about gender relations (PNUD, 2010), statements derived from SJT are not uniform across societies. It may be possible that contextual factors are moderating the palliative function of ideology, but we need samples from different countries in order to test this hypothesis. Although, SJT explicitly states the relevance of socialization processes and contextual factors (Jost et al., 2001; Jost and Hunyady, 2002), research under this theoretical approach, in general, does not account for them.

### Limitations and Future Research

The exposed results have three main limitations that we need to consider. First, the socioeconomic measure (i.e., Vulnerability School Index) was assessed at the school level. A more appropriate index is far more complicated to collect, because of the age of the students, who not always know the income or specific occupation of their parents. Nevertheless, when we considered data analyses more appropriate to the nested structure of data and variables, such as multilevel linear regression modes, we found a similar pattern of results.

The second main limitation of the study is related to the type of sexism used. In this research, we used a hostile sexism scale, which was designed specifically for this survey. However, recent theoretical and empirical advances suggest that benevolent sexism is strongly associated with aspects about system justification. An exception is the study conducted by Brandt (2011), who found that a measure of hostile sexism longitudinally predicted gender inequality in a cross-cultural sample. Although we need to conduct further research that includes assessments of benevolent sexism, comparing its effects with those of hostile sexism, the present research advanced in theoretical and empirical statements related to sexism and system justification.

Finally, our research used a theoretical approach which provides a similar rationale for the palliative function of both stereotypes and prejudice. For both constructs, SJT proposes that they function as a legitimizing ideology (e.g., Calogero and Jost, 2011; Connelly and Heesacker, 2012; Napier et al., 2010). Nevertheless, from a theoretical point of view both constructs are not equivalent (e.g., Devine, 1989; Dovidio and Gaertner, 1999; Stangor, 2009; Amodio, 2014). Although they are highly correlated and share some neural processes, they can be distinguished. For this reason, future research should decompose the unique variance explained by gender stereotypes and sexism (both hostile and benevolent sexism).

## Ethics Statement

This study was carried out in accordance with the recommendations of the International Survey of Children Well-Being Committee with written informed consent from all subjects. All subjects gave written informed consent in accordance with the Declaration of Helsinki. The protocol was approved by the International Survey of Children Well-Being Committee.

## Author Contributions

SV-S: theoretical review, data analysis, and wrote the article.

## Conflict of Interest Statement

The author declares that the research was conducted in the absence of any commercial or financial relationships that could be construed as a potential conflict of interest.
